# Adiponectin-derived active peptide ADP355 exerts anti-inflammatory and anti-fibrotic activities in thioacetamide-induced liver injury

**DOI:** 10.1038/srep19445

**Published:** 2016-01-18

**Authors:** Huafeng Wang, Huan Zhang, Zimu Zhang, Biao Huang, Xixi Cheng, Dan Wang, Zha la Gahu, Zhenyi Xue, Yurong Da, Daiqing Li, Zhi Yao, Fei Gao, Aimin Xu, Rongxin Zhang

**Affiliations:** 1 Laboratory of Immunology and Inflammation, Department of Immunology and Research Center of Basic Medical Sciences, Tianjin Medical University, Tianjin, China; 2 Department of Immunology, Tianjin Key Laboratory of Cellular and Molecular Immunology; Key Laboratory of Immune Microenvironment and Diseases, Ministry of Education of China, Tianjin Medical University, Tianjin, China; 3 School of Life Science, Shanxi Normal University, Linfen, China; 4 Department of Cell Biology, Logistic College of CAPF, Tianjin, China; 5 Key Laboratory of Hormones and Development (Ministry of Health), Metabolic Diseases Hospital and Tianjin Institute of Endocrinology, Tianjin Medical University, Tianjin 300070, China; 6 State Key Laboratory of Reproductive Biology, Institute of Zoology, Chinese Academy of Sciences, Beijing, PR China; 7 State Key laboratory of Pharmaceutical Biotechnology, and Department of Medicine, University of Hong Kong, Hong Kong, China

## Abstract

Adiponectin is an adipocyte-derived circulating protein with beneficial effects on injured livers. Adiponectin-deficient (adipo(−/−)) mice develop enhanced liver fibrosis, suggesting that adiponectin could be a therapeutic target for liver injury. In the present study, we investigated the protective role of ADP355, an adiponectin-based active short peptide, in thioacetamide (TAA)-induced acute injury and chronic liver fibrosis in mice. ADP355 remarkably reduced TAA-induced necroinflammation and liver fibrosis. ADP355 treatment increased liver glycogen, decreased serum alanine transaminase and alkaline phosphatase activity, and promoted body weight gain, hyper-proliferation and hypo-apoptosis. In addition, ADP355 administration suppressed the TAA-induced activation of hepatic stellate cells and macrophages in the liver. These were associated with the inactivation of TGF-β1/SMAD2 signaling and the promotion of AMPK and STAT3 signaling. Sensitivity of adipo(−/−) mice to chronic liver injury was decreased with ADP355. In conclusion, ADP355 could mimic adiponectin’s action and may be suitable for the preclinical or clinical therapy of chronic liver injury.

Numerous studies have analyzed the roles of adipokines in the hepatic wound healing process, identifying novel roles as modulators of liver pathophysiology^1^. As one of the adipokines, adiponectin is a circulating adipocyte-derived protein and was originally identified independently by four groups using different approaches^2^. It has been reported that adiponectin may exert protective effects against liver fibrosis. Leptin-mediated hepatic fibrosis could be disrupted with adiponectin administration^3^,^4^. Mice lacking adiponectin developed enhanced carbon tetrachloride-induced liver fibrosis^5^. A probable mechanism is that adiponectin could defend collagens to negatively regulate the growth of myelomonocytic progenitors and the functions of macrophages^6^. In addition, adiponectin is also beneficial in many other diseases, such as cardiovascular disease, insulin resistance, cancer, and inflammatory conditions^2^.

However, the development of the whole adiponectin protein as a drug is difficult because of the extreme insolubility of the C-terminal globular domain and its larger peptide fragments^7^, which may limit target therapy by using full-length adiponectin. Moreover, adiponectin exerts different biological activities in an oligomerization-dependent manner^6^. To bypass the limitations described above, some researchers began to focus efforts on developing some simpler and smaller molecules associated with adiponectin that can function as adiponectin receptor agonists to mimic adiponectin’s action. This has slowly become a reality. Recently, a novel adiponectin-based short peptide, ADP355, was generated using a panel of 66 overlapping 10-amino-acid-long peptides covering the entire adiponectin globular domain (residues 105–254)^7^. As reported, it showed anti-tumor effects on several adiponectin receptor-positive cancer cell lines in a dose-dependent manner and on the growth of orthotopic human breast cancer xenografts by 31% *in vivo*^7^. Very recently, ADP355 was reported to offset the detrimental effects of HIV protease inhibitor (PI)-induced metabolic dysfunction in experimental animal models^8^. These results showed that ADP355 administration partially reversed PI-induced loss of subcutaneous adipose tissue; attenuated PI-induced hyperinsulinemia, hypertriglyceridemia, and hypoadiponectinemia; and prevented PI-induced cognitive impairment and brain injury^8^. ADP355 was also used as a positive control for screening adiponectin receptor agonists in a high-throughput assay based on a fluorescence polarization, and several compounds were identified as potential adiponectin receptor agonists^9^.

Based on the above-described results, we proposed the hypothesis that as an adiponectin receptor agonist, ADP355 could reduce liver injury in mice. In this study, we established the experimental liver injury models with TAA, and the administration of ADP355 was performed to examine whether ADP355 functions with adiponectin-like action against inflammation and liver fibrosis and attenuated the enhancement of liver fibrosis in adipo(−/−) mice.

## Results

### ADP355 Inhibits the Progression of TAA-Induced Liver Fibrosis

**Figure 1:**
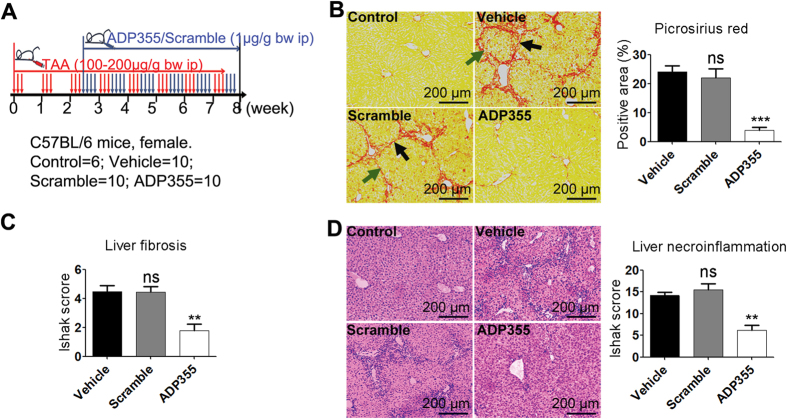
ADP355 ameliorates TAA-induced liver fibrosis in mice. (**A**) Schedule of fibrosis induction and ADP355 therapy. Control = no treatment; vehicle = TAA + PBS; scramble = TAA + scramble; ADP355 = TAA + ADP355. (**B**) Liver fibrosis was determined using picrosirius red staining. As shown by arrows, the obvious fibroplasias extended from the central venous or portal areas to the surrounding area with marked P-P (portal to portal, black arrows) and P-C (portal to central, green arrows) bridging due to induced TAA in the vehicle mice. (**C**) Staging of liver fibrosis. (**D**) Necroinflammation was analyzed by H&E, respectively. Histopathological evaluations were conducted using Ishak’s scoring system and Image-Pro Plus 6 Windows Software. Representative images are shown for all panels. Mean ± SD; n = 4–6 samples/group. Compared to the control or vehicle group, *p < 0.05, **p < 0.01, ***p < 0.001 were calculated using two-tailed Student’s t test (T TEST).

Liver fibrosis is a characteristic of every chronic liver disease^10^. PSR staining for liver samples showed that the obvious fibroplasias extended from the central venous or portal areas to the surrounding area with marked P-P (portal to portal) and P-C (portal to central) bridging due to induced TAA in the vehicle mice (Fig. 1B,C). By contrast, the ADP355-treated group exhibited remarkably reduced fibrosis, while almost no changes were observed in the scramble-treated group (Fig. 1B,C). The negative effects of ADP355 on liver fibrosis were further confirmed by quantified staining and pathologic score. H&E staining demonstrated that liver necroinflammation caused by TAA was reversed successfully in ADP355-treated mice, but this reversal was unsuccessful in scramble mice (Fig. 1D).

### ADP355 Recovers Mice from Liver Dysfunction in Chronic Injury by Enhancing Liver Regeneration and Reducing Liver Apoptosis

**Figure 2:**
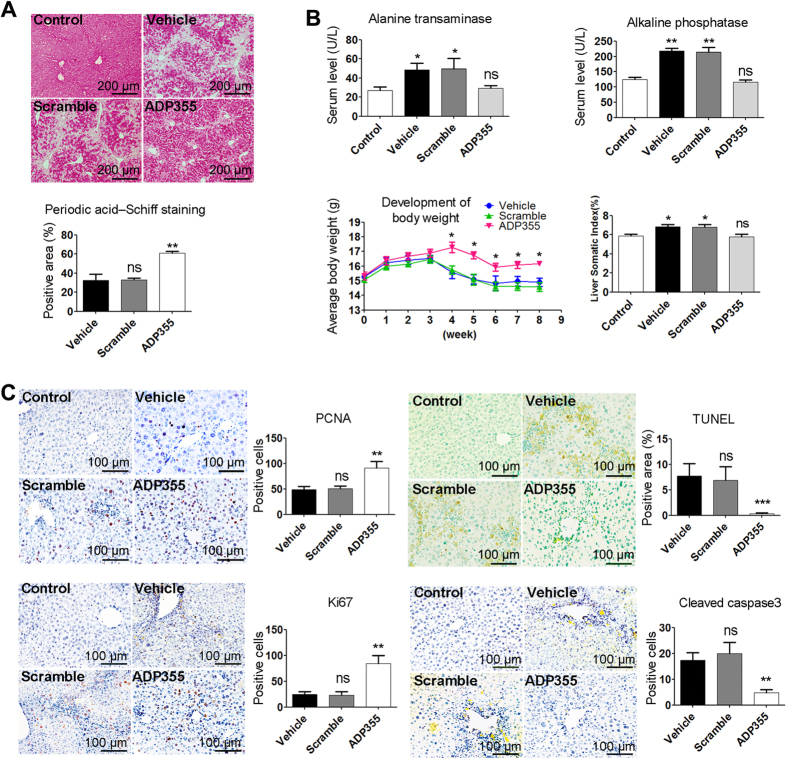
ADP355 ameliorates TAA-induced liver fibrosis in mice. (**A**) Glycogen were analyzed by periodic acid–Schiff staining. (**B**) Liver functional analysis for alanine transaminase, alkaline phosphatase and body weight gain.Liver somatic index (LSI) = liver weight/body weight (%). (**C**) Liver regeneration analysis for PCNA, TUNEL, Ki67 and cleaved caspase 3. Representative images are shown. Histopathological evaluations were conducted using manual method and Image-Pro Plus 6 Windows Software. Mean ± SD; n = 4–6 samples/group. Compared to the control or vehicle group, *p < 0.05, **p < 0.01, ***p < 0.001 were calculated using two-tailed Student’s t test (T TEST).

As shown, ADP355-treated mice showed increased liver glycogen (PAS staining, Fig. 2A), decreased serum alanine transaminase and alkaline phosphatase activity, body weight gain and decreased liver somatic index compared the vehicle and scramble mice (Fig. 2B). In the development of liver fibrosis, body weight loss in company with increased liver somatic index might be caused by liver dysfunction. ADP355-treated mice showed higher body weight and lower liver somatic index compared to the vehicle and scramble mice (Fig. 2B). Staining for PCNA, TUNEL, Ki67 and cleaved caspase-3 showed that ADP355 treatment was followed by significant hyper-proliferation compared with only TAA (vehicle), while liver apoptosis was remarkably reduced by ADP355 treatment (Fig. 2C).

### ADP355 Suppressed the Activation of Hepatic Stellate Cells Induced by TAA

**Figure 3:**
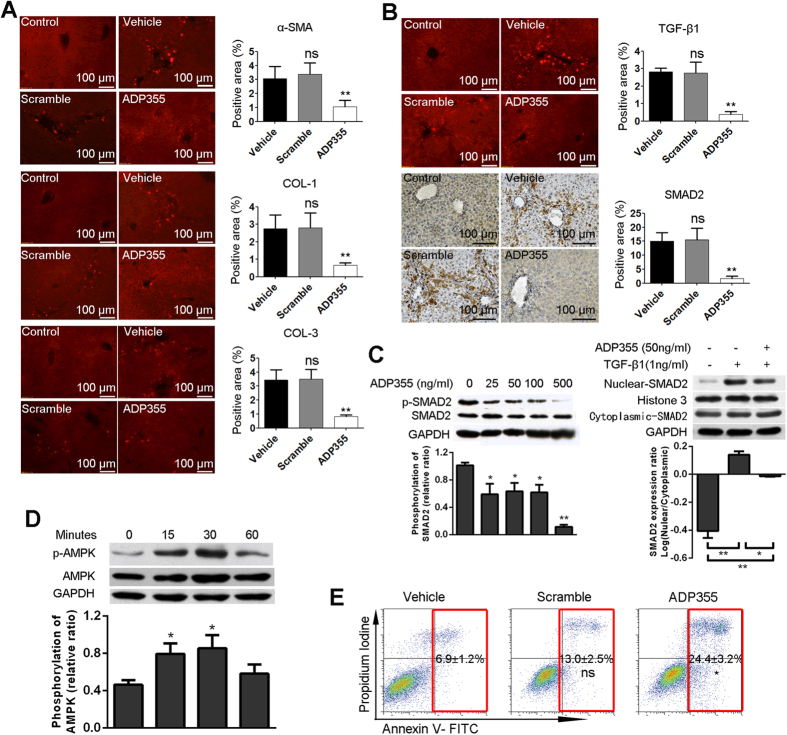
ADP355 inhibits the activation of hepatic stellate cells. (**A**) Inactivation of hepatic stellate cells by ADP355 *in vivo* is investigated by immuno-staining for α-SMA, COL-1 and COL-3 with liver sections. (**B**) TGF-β1/SMAD2 signaling *in vivo* is examined by immuno-staining for TGF-β1 and SMAD2 with liver sections. (**C**) LX-2 cells (hepatic stellate cells) were treated with ADP355 (0–500 ng/ml) in the presence of TGF-β1 (1 ng/mL) for 40 minutes; the phosphorylation and nuclear translocation of SMAD2 with the indicated dose of ADP355 were all examined. Bar graphs show densitometric analysis of intensities of (left) p-SMAD2/SMAD2 relative to that of GAPDH and (right) nuclear/cytoplasmic-SMAD2 relative to that of Histone 3/GAPDH. (**D**) ADP355 increases the phosphorylation of AMPK in LX-2 cells. The protein levels of phosphor-AMPK and AMPK are determined by Western blot analysis. Bar graph shows densitometric analysis of intensities of p-AMPK/AMPK relative to that of GAPDH. (**E**) The apoptosis of LX-2 cells is induced by ADP355. Representative images are shown and histopathological evaluations were conducted using Image-Pro Plus 6 Windows Software. Mean ± SD; n = 4–6 samples/group. In comparison to the vehicle group, *p < 0.05, **p < 0.01, ***p < 0.001 were calculated using two-tailed Student’s t test (T TEST).

Activated HSCs (hepatic stellate cells) play an important role in producing ECM (extracellular matrix) that leads to fibrogenesis^11^. As shown, the expression of α-SMA (marked as the activation of HSC) and the production of ECM (marked as type I collagen and type III collagen) were downregulated significantly after ADP355 treatment (Fig. 3A). Activation of HSCs requires the activation of the TGF-β1/SMAD signaling pathway^12^. Our *in vivo* studies showed that the administration of ADP355 decreased the levels of TGF-β1 and SMAD2 proteins in the liver (Fig. 3B), while *in vitro* LX-2 cells treated with ADP355 resulted in significant up-expression of adiponectin receptors, the inhibition of SMAD2/3 phosphorylation and SMAD2 nuclear translocation (Fig. 3C, Supplementary Figs S1, S2). Multiple effects of adiponectin, especially in the liver, are known to be derived from the activation of adenosine monophosphate-activated protein kinase (AMPK)^3^,^13^,^14^,^15^,^16^. It has been reported that adiponectin induces apoptosis of activated HSCs through AMPK activation^17^,^18^. Increased AMPKα phosphorylation was detected at 30 min in the LX-2 cells stimulated with ADP355 (Fig. 3D). Moreover, LX-2 cell apoptosis was induced by ADP355 (50 ng/ml) over 24 hours (Fig. 3E).

### ADP355 Blocks the Activation of Macrophages by Promoting STAT3 Signaling

**Figure 4:**
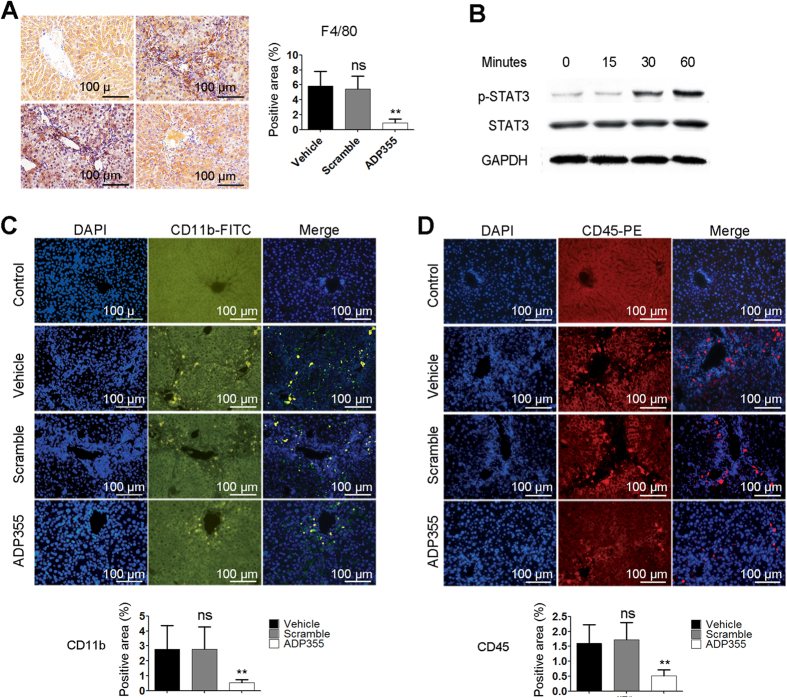
ADP355 negatively regulates macrophage activation. (**A**) Inactivation of macrophages by ADP355 is indicated by immune-staining for F4/80 with liver sections. (**B**) Western blotting of p-STAT3 in RAW 264.7 cells (macrophages). (**C,D**) Inflammatory cells in the liver are further determined with CD11b and CD45. Representative images are shown and histopathological evaluations were conducted using Image-Pro Plus 6 Windows Software. Mean ± SD; n = 4–6 samples/group or three independent experiments. In comparison to vehicle group, *p < 0.05, **p < 0.01, ***p < 0.001 were calculated using two-tailed Student’s t test (T TEST).

It has been reported that adiponectin may act as soluble defense collagen by negatively regulating the functions of macrophages^6^. STAT3 is a potential negative regulator of inflammatory responses by suppressing activation of macrophage^19^,^20^,^21^. As shown, ADP355 treatment strikingly suppressed the activation of hepatic macrophages (marked as F4/80) induced by TAA *in vivo* (Fig. 4A) and promoted the phosphorylation of STAT3 in RAW 264.7 cells (macrophages) *in vitro* (Fig. 4B). Mononuclear CD45- and CD11b-positive leukocyte infiltration into the liver was also significantly attenuated after ADP355 treatment *in vivo* (Fig. 4C,D). In the *in vivo* studies, flow cytometry analysis of helper T cells in spleens showed that ADP355 treatment reduces the proliferation of helper T cells mediated by macrophages in TAA-induced liver injury (Supplementary Fig. S3).

### Administration of ADP355 Decreases the Sensitivity of Adiponectin-Deficient Mice to Chronic Liver Injury

**Figure 5:**
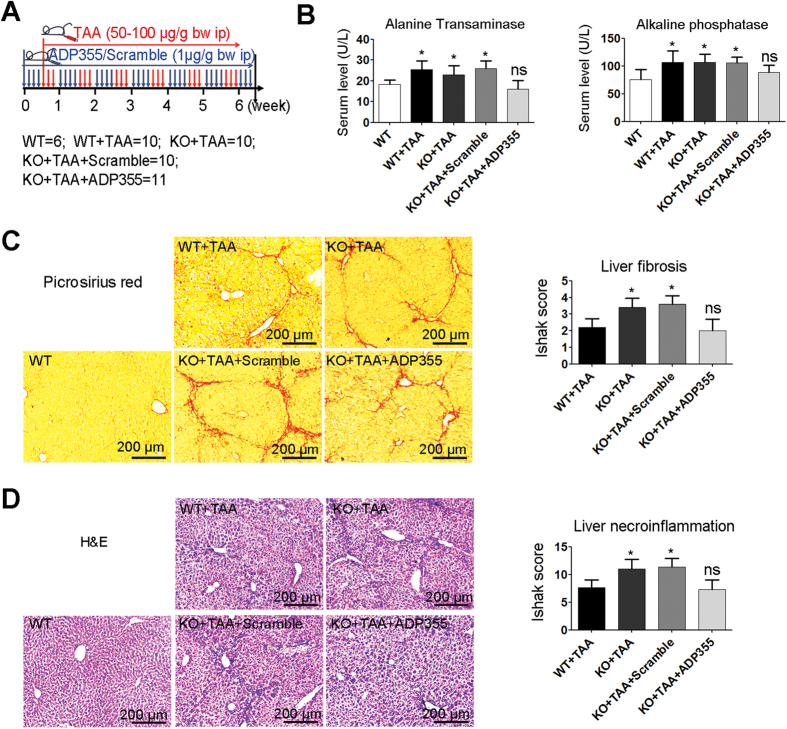
ADP355 attenuates TAA-induced chronic liver fibrosis in adiponectin-deficient mice. (**A**) Schedule of fibrosis induction and ADP355 therapy. (**B**) Serum ALP and ALT. (**C**,**D**) Liver fibrosis and necroinflammation are analyzed by picroirius red and H&E staining. Representative images are shown and histopathological evaluations were conducted using Ishak’s scoring system. Mean ± SD; n = 4–6 samples/group. Compared to the WT or WT + TAA group, *p < 0.05, **p < 0.01, ***p < 0.001 are calculated using two-tailed Student’s t test (T TEST).

It was reported that adipo(−/−) mice (KO) were vulnerable to CCl4-induced hepatic fibrosis^5^. Therefore, we evaluated the therapeutic potential of ADP355 in mice that are genetically defective in adiponectin (Fig. 5A). Administration of ADP355, but not scramble, decreased the serum ALT/ALP levels and the sensitivity of adipo(−/−) mice to TAA-induced liver fibrosis and necroinflammation (Fig. 5B–D).

**Figure 6:**
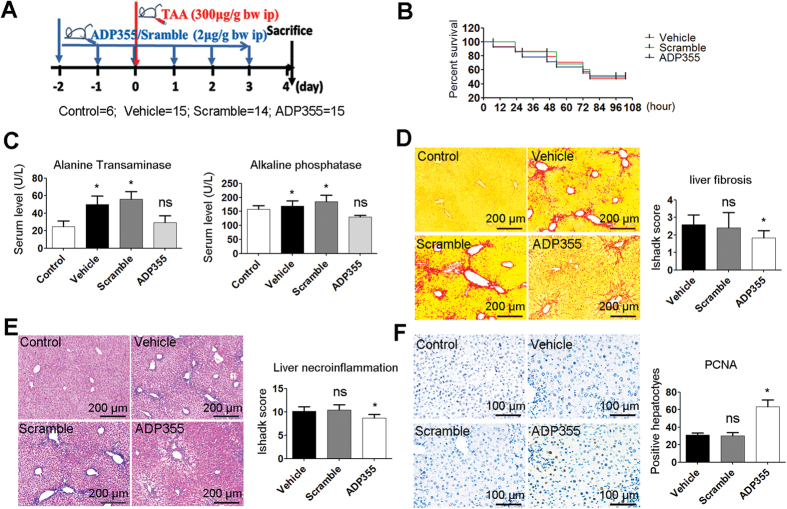
ADP355 decreases TAA-induced acute liver injury. (**A**) Schedule of acute liver injury induction and ADP355 therapy. (**B**) Kaplan-Meier survival analysis of mice. (**C**) Serum ALP and ALT. (**D**,**E**) Liver samples from live mice are analyzed by picrosirius red and H&E. Pathologic scores of liver biopsies determined by Ishak system. (**F**) Immuno-staining for PCNA. Histopathological evaluations were conducted using manual method. Representative images are shown for all panels. Mean ± SD; n = 4–6 samples/group. *p < 0.05, **p < 0.01, ***p < 0.001 are calculated using two-tailed Student’s t test (T TEST).

In addition, we also examined the protective effect of ADP355 on acute liver injury (Fig. 6A). Statistical analyses revealed that the difference in survival with TAA treatment between the ADP355 and the vehicle was not significant (Fig. 6B). However, 4 days after TAA administration, the ALT and ALP levels of the ADP355-treated mice were significantly lower than those of the vehicle mice (Fig. 6C). The ADP355-treated mice showed reduced liver fibrosis and necroinflammation and showed greater hyper-proliferation (Fig. 6D–F).

## Discussion

As a beneficial hormone molecule, adiponectin has been reported to exhibit protective effects against liver injury^1^,^3^,^4^. However, adiponectin is made up of 244 amino acids and is a relatively larger cytokine, which restricts its clinical applications because it is difficult for drug manufacturers to purify the full-size adiponectin protein for mass production. ADP355, an adiponectin-based short peptide, was designed and developed by Otvos L *et al*., who demonstrated its initial preclinical development, acting as an AdipoR agonist in cancer cells^7^. Importantly, the peptide displayed excellent stability in mouse serum and blood and a favorable toxicity profile^7^. In the present study, we provide the first clear evidence that the delivery of ADP355 has a similar therapeutic potential to adiponectin in dramatically reducing liver injury induced by TAA.

The liver has evolved a unique capacity for regeneration so that the organ can be rapidly and efficiently repaired for the maintenance of its important functions after injury^22^,^23^. Nevertheless, during the progression of many liver diseases (especially in advanced liver fibrosis), increasingly extensive fibrogenesis impedes liver regeneration and then leads to compromised liver function^23^. Therefore, it is important for the clinical therapy of liver diseases to stimulate liver regeneration and accelerate the restoration of liver structure and function. There is already evidence that adiponectin *in vivo* is implicated in the normal progress of liver regeneration. The 2/3 partial hepatectomy (PH) in adiponectin-deficient mice, compared to wild-type mice, showed impaired liver mass re-growth and decreased hepatocyte proliferation^24^. The stimulation of liver regeneration by adiponectin was re-enacted with ADP355 in our study. The results demonstrated that the administration of ADP355 remarkably increased the number of proliferating hepatocytes in the injured liver (marked as PCNA and Ki67) and inhibited the apoptosis of the liver (marked as TUNEL and cleaved caspase-3). Thus, mice that recovered from liver dysfunction were shown to exhibit body weight loss, higher serum ALT/ALP and lower liver glycogen.

Liver fibrosis is the result of wound healing in response to liver tissue injury (especially persistent injury). Growing evidence indicates that liver fibrosis could be reversible, and even the advanced cirrhosis phenotype is now considered potentially reversible^25^. A critical contributor to liver fibrosis is the generation and proliferation of α-smooth muscle actin-positive myofibroblasts from periportal and perisinusoidal areas. It is considered that HSCs have a fibrogenic potential and are one major source of hepatic myofibroblasts. HSCs are quiescent in the normal liver, reside in the space of Disse and store vitamin A in lipid droplets. In response to liver damage, quiescent HSCs are activated to lose vitamin A and transdifferentiate into a myofibroblast phenotype, expressing α-smooth muscle actin. These cells have a high proliferative index and are the major source of fibrillar collagen^26^. As a profibrogenic cytokine, leptin increases HSC proliferation and inhibits HSC apoptosis^27^. In contrast to leptin, adiponectin promotes HSC apoptosis, blocks HSC proliferation, and protects the liver from fibrosis^17^. Adipo(−/−) mice developed enhanced carbon tetrachloride-induced liver fibrosis^5^. One of the protective effects of liver fibrosis for adiponectin is as an antagonist of leptin to inhibit the activation of HSC^3^,^4^. In our study, ADP355 treatment remarkably reduced liver fibrosis. Further studies showed that the expression of α-SMA in the liver from ADP355-treated mice, compared to vehicle mice, decreased significantly, indicating that TAA injury-induced HSC proliferation could be significantly suppressed by ADP355. Meanwhile, compared to those of the Vehicle group, the production of type 1 collagen and type 3 collagen in the livers of ADP355-treated mice was also significantly reduced. In addition, ADP355 promoted the apoptosis of HSC LX-2 *in vitro*.

TGF-β is the potent profibrogenic mediator of liver fibrosis^12^. The causative roles of TGF-β1 in the activation of HSCs and hepatic fibrogenesis have been shown using transgenic knockout mice and adenoviral overexpression^28^,^29^. The activation of HSCs increases the responsiveness to TGF-β1 through the upregulation or de novo expression of the three TGF-β1 receptors (types I, II, and III) and activates its downstream SMAD signaling pathways; thereby, phosphorylated SMAD2 and SMAD3 translocate to the nucleus, regulating gene transcription^11^. As an inhibitor of TGF-β1, nilotinib successfully reduced the CCL4 and bile duct ligation-induced liver fibrosis^30^. Furthermore, TGF-β1 and its activators are also potent inhibitors of the growth of hepatocytes *in vitro* and in regenerating the liver *in vivo*, where it may serve as the terminator of the replicative response to partial hepatectomy^12^. In addition to blocking cell growth in the liver, TGF-β also induces apoptosis *in vitro* in cultured hepatocytes^31^. Overexpression of TGF-β *in vivo* would cause high levels of hepatocyte apoptosis^28^. Leptin promotes profibrogenic responses in the liver in part by up-regulating TGF-β1^32^. The activation and proliferation of hepatic stellate cells induced by TGF-β1, leptin and others can be lowered by the pharmacological activation of AMPK stimulated by adiponectin^3^,^16^,^17^, subsequently blocking the secretion of TIMP-1 and significantly increasing MMP-1 activity^3^.

In our studies, ADP355 treatment decreased the expression of TGF-β1 and SMAD2 in the TAA-damaged liver. SMAD2 phosphorylation and nuclear translocation were dampened, while AMPK phosphorylation was enhanced with ADP355 treatment *in vitro*. At the same time, TGF-β1 is a potent inhibitor of the growth of hepatocytes *in vitro* and in regenerating liver *in vivo*, where it may serve as the terminator of the replicative response to partial hepatectomy. Therefore, the suppression of ADP355 on TGF-β1/SMAD2 signal through the activation of AMPK inhibits not only fibrosis but also liver apoptosis.

Macrophages are found in close proximity to collagen-producing myofibroblasts and indisputably play a key role in fibrosis. They produce profibrotic mediators such as TGF-β1 and control extracellular matrix turnover by regulating the balance of various matrix metalloproteinases and tissue inhibitors of matrix metalloproteinases^33^. Their number increases in the damaged liver, and they are principally located around the regions of damage and fibrosis^25^,^34^. The presence of a small amount of macrophages can promote the rapid transformation of hepatic stellate cells from quiescent to active *in vitro*^35^. Blockade of macrophage infiltration inhibits the activation of hepatic stellate cells and leads to the suppression of liver fibrogenesis^36^. GdCl3 treatment suppresses HSC activation by selectively diminishing Kupffer, reducing liver fibrosis^37^. The anti-inflammatory effects of adiponectin are closely related to its inhibition of macrophage function. Adiponectin can normalize Toll-like receptor-4 (TLR-4)-mediated signaling in hepatic macrophages after ethanol feeding, likely contributing to the hepatoprotective effect of adiponectin in the progression of alcoholic liver disease^38^. The treatment of cultured macrophages with adiponectin significantly inhibited their phagocytic activity and their lipopolysaccharide-induced production of tumor necrosis factor^6^. Importantly, overnight treatment of Kupffer cells with adiponectin can normalize the chronic ethanol-induced sensitization of LPS-stimulated TNF-α expression, normalizing the increased production of ROS^6^,^39^,^40^. The anti-inflammatory effects of adiponectin are associated with increased expression of anti-inflammatory mediators, such as interleukin (IL)-10^41^, which is regulated by the transcription factor STAT3, a potential negative regulator of inflammatory responses^19^,^42^. Blockade of STAT3 promotes macrophage activation^19^,^20^. The pro- and anti-inflammatory role of STAT3 is cell type-dependent in liver injury; in hepatocytes, STAT3 promotes inflammation, whereas in macrophages/Kupffer cells, it suppresses inflammation^43^. However, macrophages/Kupffer cells play a major role in inducing inflammation in the liver, and the anti-inflammatory effect of STAT3 in macrophages/Kupffer cells likely dominates the proinflammatory effect in hepatocytes^43^. STAT3 activation also promotes liver regeneration, and knockdown of STAT3 dampened the division and proliferation of hepatocytes and enhanced hepatocellular damage in mouse liver ischemia/reperfusion injury^20^ or after partial hepatectomy^44^. Adiponectin deficiency impairs liver regeneration by attenuating STAT3 phosphorylation in mice^24^. In our study, we observed that the activity of macrophages in the liver was significantly decreased and the infiltration of other populations of immune cells into the liver was notably suppressed with ADP355 treatment in TAA-induced liver injury. STAT3 phosphorylation was enhanced in RAW 264.7 macrophages with ADP355 treatment.

Taken together, our results have shown that ADP355 can mimic the actions of adiponectin to exert a therapeutic effect on TAA-induced liver injury (Supplementary Fig. S4). It can promote liver regeneration and inhibit liver apoptosis, HSC-activated fibrosis and macrophage-mediated inflammation and then restore the liver from dysfunction. These actions are at least partially attributable to the activation of AMPK or STAT3 signaling and suppression of TGF-β1/SMAD2 signaling by ADP355. As a simple molecule that is easy to synthesize, it is expected that ADP355 shows promise in adiponectin-associated clinical applications.

## Methods

### Chemicals and Animals

Thioacetamide (TAA) (Sigma-Aldrich, Switzerland), ADP355 (H-Asn-Ile-Pro-Nva-Leu-Tyr-Ser-Phe-Ala- DSer-NH2) and scramble (H-Pro-Ile-Asn-Tyr-Ala-Nva-Ser-Phe-Leu-Ser-NH2) (Lysine Bio-system, China) were all dissolved in PBS. C57BL/6 mice (from Academy of Military Medical Science, Beijing, China) and adipo(−/−) mice (Aimin Xu, The University of Hong Kong). All experimental protocols were approved by Tianjin Medical University Animal Ethics Committee and all methods were carried out in accordance with the approved guidelines.

### Liver injury

To investigate the anti-fibrogenic effects of ADP355 (Fig. 1A), female mice (8 weeks, 15–16 g) were randomly divided into four groups: (i) control (n = 6) with PBS alone, (ii) vehicle (n = 10) with TAA + PBS, (iii) ADP355 (n = 10) with TAA + ADP355, (iv) scramble (n = 10) with TAA + scramble. Chronic liver injury was induced by TAA (100 μg/g bw ip) three consecutive times per week. ADP355/scramble (1 μg/g bw ip) was added after nine administrations of TAA. Eight weeks later, all animals were sacrificed, and tissue was harvested 3 days after the last TAA injection. Body weights of the mice were recorded once a week.

To investigate the compensatory substitute of ADP355 for adipo(−/−) mice in chronic liver injury (Fig. 4A), male mice (10 weeks, 22–25 g) were randomly divided into five groups, (i) WT (n = 6): wild type mice with PBS alone, (ii) WT + TAA (n = 10): wild type mice with TAA, (iii) KO + TAA(n = 10): adipo(−/−) mice with TAA, (iv) KO + TAA + Scramble (n = 11): adipo(−/−) mice with TAA + scramble, and (v) KO + TAA + ADP355: adipo(−/−) mice with TAA + ADP355. Other than the experiment above, ADP355/scramble was treated before TAA administration and ended 6 weeks later.

### ALT and ALP

Serum samples were collected from eyeball blood in anesthetized mice. Alanine transaminase (ALT) and alkaline phosphatase (ALP) activities were determined using an EnzyChrom^TM^ Alanine Transaminase Assay Kit and QuantiChrom^TM^ Alkaline Phosphatase Assay Kit (BioAssay Systems), respectively.

### Histology

Livers were perfused with PBS and then removed, weighed, and cut into pieces (0.5 cm × 0.5 cm). Liver pieces were embedded in paraffin after being fixed in 4% (weight/volume) paraformaldehyde and were cut into 6-μm-thick sections. Paraffin sections were stained with hematoxylin–eosin (H&E) for inflammation and necrosis, Picrosirius red (PSR) for fibrosis and periodic acid-Schiff (PAS) for glycogen stores. Necroinflammatory scoring with H&E staining and staging of liver fibrosis with PSR staining were adapted from a modified Ishak system (Supplementary Table S1 and Table S2)^11^,^45^. Staging of liver fibrosis is defined as follows: 0, no fibrosis; 1, some portal tract fibrotic ± short fibrous septa; 2, most portal tract fibrotic ± short fibrous septa; 3, most portal tract fibrotic with occasional P-P bridging; 4, portal tract fibrotic with marked P-P and P-C bridging; 5, marked P-P and/or P-C bridging with occasional nodules (incomplete cirrhosis); 6, cirrhosis (probable or definite). Necroinflammatory scores = A + B + C + D. A (piecemeal necrosis) scores are defined as follows: 0, absent; 1, mild (focal, few portal areas); 2, mild/moderate (focal, most portal areas); 3, moderate around less than 50% of tracts or septa/P-P bridging necrosis); 4, severe (continuous around more than 50% of tracts or septa/P-P bridging necrosis). B (confluent necrosis) scores are defined as follows: 0, absent; 1, focal confluent necrosis; 2, zone 3 necrosis in some areas; 3, zone 3 necrosis in most areas; 4, zone 3 necrosis, plus occasional portal-central (P-C) bridging; 5, zone 3 necrosis, plus multiple P-C bridging; 6, panacinar or multiacinar necrosis. C (focal lytic necrosis, apoptosis and focal inflammation scores are defined as follows: 0, absent; 1, one focus or less per 10x objective (ob); 2, two to four foci per 10x ob; 3, five to ten foci per 10x ob; 4, more than ten foci per 10x ob. D (portal inflammation) scores are defined as follows: 0, none; 1, mild, some or all portal areas; 2, moderate, some or all portal areas; 3, moderate/marked, all portal areas; 4, marked, all portal areas.

### Immunostaining

After deparaffinization and rehydration of the liver sections, antigen retrieval was performed under high temperature and pressure in 0.01 M citrate buffer (pH 6.0). For immunohistochemistry, the sections were then incubated with 0.3% hydrogen peroxide in methanol for 20 min at room temperature to quench endogenous peroxidase activity. After washing with PBS and blocking, the tissue sections were incubated with the following primary antibodies (1:100–500): α-SMA (Abcam), collagen I (Abcam), collagen III (Abcam), TGF-β1 (Sigma-Aldrich), SMAD2 (Sigma-Aldrich), PCNA (Cell Signaling Technology), F4/80 (AbDserotec, Oxford, UK), CD45 (eBioscience, PE), and CD11b (eBioscience, FITC). After washing, the sections were incubated with horseradish peroxidase (HRP)-conjugated secondary antibodies (1:1000; Jackson, distributed by MultiSciences Biotech Co., Ltd) specific to the species of the primary antibodies for immunohistochemistry or Alexa 555-conjugated secondary antibodies (1:200; Molecular Probes) for immunofluorescence staining. The sections were counterstained with 4,6-diamidino-2-phenylindole (DAPI) (eBioscience) or developed using 3,3’-diaminobenzidine (DAB) and counterstained with hematoxylin.

### Western Blotting for Signaling Analysis

For TGF-β1/SMAD2 signaling, LX-2 cells (hepatic stellate cells) were treated with ADP355 (0–500 ng/ml) in the presence of TGF-β1 (1 ng/mL) for 40 minutes, and the phosphorylation and nuclear translocation of SMAD2 with the indicated dose of ADP355 were all examined. The signaling of AMPK in LX-2 cells and STAT3 in RAW 264.7 (mouse macrophages) affected by the indicated ADP355 dose were also investigated.

The total proteins were extracted with RIPA Lysis Buffer (Beijing Biomed) composed of the protease inhibitor PMSF, and the nuclear proteins were extracted with the Nucleoprotein Extraction Kit (Sangon Biotech, Shanghai). The cell lysates were separated by SDS–PAGE and transferred to Immobilon-P membranes (Millipore). After incubation with primary antibodies for p-AMPKα (Cell Signaling), AMPKα (Cell Signaling), p-STAT3 (Cell Signaling), p-SMAD2 (cell signaling technology), GAPDH (Santa Cruz biotechnology), and anti-histone 3.1 (Tianjin Sungene Biotech) at 4 °C overnight with gentle shaking and washing with Tris-buffered saline (50 mM Tris-HCl (pH 7.5) and 150 mM NaCl) containing 0.1% Tween-20, the membranes were incubated with HRP-conjugated secondary antibodies (Tianjin Sungene Biotech) specific to the species of the primary antibodies for 2 h at room temperature. The intensity of specific protein bands was quantified by the Quantity One 1-D Analysis Software Version 4.6.2 (Bio-Rad Laboratories).

### Apoptosis

Liver apoptosis analysis used the TACS•XL® DAB in situ Apoptosis Detection Kit (Trevigen) according to the manufacturer’s instructions. Using the Annexin V Cell Apoptosis Analysis Kit (Sungene Biotech), apoptotic LX-2 cells were analyzed using a FACScalibur (BD Biosciences, USA), and the acquired data were analyzed using Flow Jo 7.6 software (Tree Star, Inc, USA).

### Statistical Analysis

The positive area of all stained liver sections was quantified by Image-Pro Plus 6 Windows Software (Media Cybernetics, USA). The positive cells were counted manually. Data from different groups were shown as the means ± SD (standard deviation). Statistical significances between the means were determined using a two-tailed Student’s t test. Significance was considered when p ≤ 0.05. The graphs were finished using GraphPad Prism Version 5.0 (GraphPad Software Inc., San Diego CA).

## Additional Information

**How to cite this article**: Wang, H. *et al*. Adiponectin-derived active peptide ADP355 exerts anti-inflammatory and anti-fibrotic activities in thioacetamide-induced liver injury. *Sci. Rep.* **6**, 19445; doi: 10.1038/srep19445 (2016).

## Supplementary Material

Supplementary Information
