# Corrigendum: Adiponectin-derived active peptide ADP355 exerts anti-inflammatory and anti-fibrotic activities in thioacetamide-induced liver injury

**DOI:** 10.1038/srep26729

**Published:** 2016-05-31

**Authors:** Huafeng Wang, Huan Zhang, Zimu Zhang, Biao Huang, Xixi Cheng, Dan Wang, Zha la Gahu, Zhenyi Xue, Yurong Da, Daiqing Li, Zhi Yao, Fei Gao, Aimin Xu, Rongxin Zhang

Scientific Reports 6: Article number: 1944510.1038/srep19445; published online: 01182016; updated: 05312016

This Article contains typographical errors in the Methods section under subheading ‘Chemicals and Animals’,

“Thioacetamide (TAA) (Sigma-Aldrich, Switzerland), ADP355 (H-Asn-Ile-Pro-Nva-Leu-Tyr-Ser-Phe-Ala- DSer-NH2) and scramble (H-Pro-Ile-Asn-Tyr-Ala-Nva-Ser-Phe-Leu-Ser-NH2) (Lysine Bio-system, China) were all dissolved in PBS”.

should read:

“Thioacetamide (TAA) (Sigma-Aldrich, Switzerland), ADP355 (H-DAsn-Ile-Pro-Nva-Leu-Tyr-DSer-Phe-Ala- DSer-NH2) and scramble (H-Pro-Ile-Asn-Tyr-Ala-Nva-Ser-Phe-Leu-Ser-NH2) (Lysine Bio-system, China) were all dissolved in PBS”.

