# Supplementary material for: Adiponectin-derived active peptide ADP355 exerts anti-inflammatory and anti-fibrotic activities in thioacetamide-induced liver injury
Source: Sci Rep. 2016 Jan 18;6:19445. doi: 10.1038/srep19445 (PMC4725969; doi:10.1038/srep19445)
Supplement: Supplementary Information [file srep19445-s1.doc]

**Adiponectin-derived active peptide ADP355 exerts anti-inflammatory and anti-fibrotic activities in thioacetamide-induced liver injury**

Huafeng Wang^a,b,c,†^, Huan Zhang^a,†^, Zimu Zhang ^a,†^, Biao Huang^a^, Xixi Cheng^a^, Dan Wang^a^, Zha la Gahu^d^, Zhenyi Xue^a,b^, Yurong Da^a,b^, Daiqing Li^e^, Zhi Yao^b^, Fei Gao^f^, Aimin Xu^g^,* and Rongxin Zhang^a,b,d^,*

**Supplementary table S**1. Necroinflammatory Scores System (Ishak, et al, 1995)

| A | Score | Pathology |
| --- | --- | --- |
| Periportal or periseptal interface hepatitis (piecemeal necrosis) | 0 | Absent |
|  | 1 | Mild (focal, few portal areas) |
|  | 2 | Mild/moderate (focal, most portal areas) |
|  | 3 | Moderate around less than 50% of tracts or septa /P-P bridging necrosis) |
|  | 4 | Severe (continuous around more than 50% of tracts or septa /P-P bridging necrosis) |
| B | Score | Pathology |
| Confluent necrosis | 0 | Absent |
|  | 1 | Focal confluent necrosis |
|  | 2 | Zone 3 necrosis in some areas |
|  | 3 | Zone 3 necrosis in most areas |
|  | 4 | Zone 3 necrosis, plus occasional portal-central (P-C) bridging |
|  | 5 | Zone 3 necrosis, plus multiple P-C bridging |
|  | 6 | Panacinar or multiacinar necrosis |
| C | Score | Pathology |
| Focal (spotty) lytic necrosis, apoptosis and focal inflammation | 0 | Absent |
|  | 1 | One focus or less per 10× objective (ob) |
|  | 2 | Two to four foci per 10× ob |
|  | 3 | Five to ten foci per 10× ob |
|  | 4 | More than ten foci per 10× ob |
| D | Score | Pathology |
| Portal inflammation | 0 | None |
|  | 1 | Mild, some or all portal areas |
|  | 2 | Moderate, some or all portal areas |
|  | 3 | Moderate/marked, all portal areas |
|  | 4 | Marked, all portal areas |
| Necroinflammatory scores=A+B+C+D | | |

**Supplementary table S**2. Ishak index for staging of liver fibrosis

| Score | pathology |
| --- | --- |
| 0 | No fibrosis |
| 1 | Some portal tract fibrotic ± short fibrous septa |
| 2 | Most portal tract fibrotic ± short fibrous septa |
| 3 | Most portal tract fibrotic with Occasional P-P bridging |
| 4 | Portal tract fibrotic with marked P-P and P-C bridging |
| 5 | Marked P-P and/or P-C bridging with occasional nodules (incomplete cirrhosis) |
| 6 | Cirrhosis (probable or definite) |

**Supplementary table S3. Primers for PCR with LX-2 cells.**

| gene |  |  |
| --- | --- | --- |
| Homo sapiens GAPDH | Forward Primer | GGAGCGAGATCCCTCCAAAAT |
|  | Reverse Primer | GGCTGTTGTCATACTTCTCATGG |
| Homo sapiens ADIPOR1 | Forward Primer | ACGTTGGAGGGTCATCCCATA |
|  | Reverse Primer | AAACAGCACGAAACCAAGCAG |
| Homo sapiens ADIPOR2 | Forward Primer | CTGGATGGTACACGAAGAGGT |
|  | Reverse Primer | TGGGCTTGTAAGAGAGGGGAC |

**
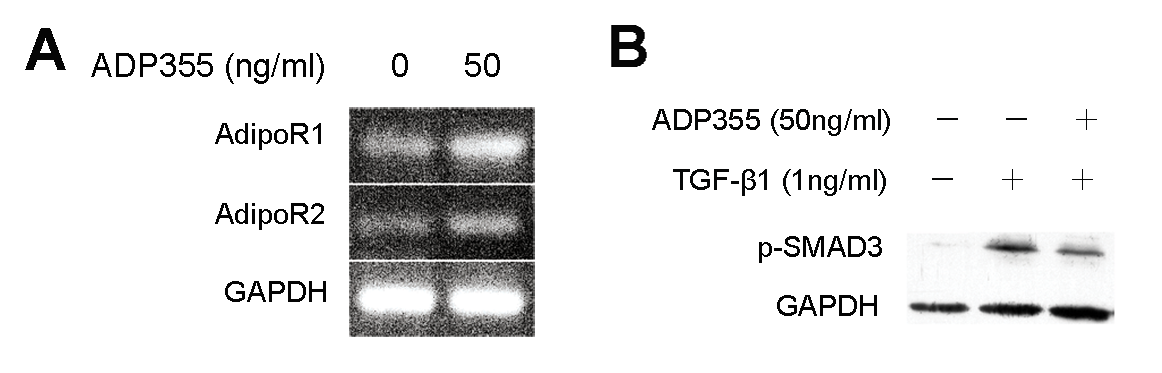
**

**Supplementary figure S1.** Effects ADP355 of on the mRNA expression of adiponectin receptors (AdipoR1 and AdipoR2) and the phosphorylation of SMAD3. (A) ADP355 promotes the mRNA expression of adiponectin receptors (AdipoR1 and AdipoR2). (B) Effects of ADP355 on the phosphorylation of SMAD3 in LX-2 cells stimulated with TGF-β1. Representative images are shown for all panels.


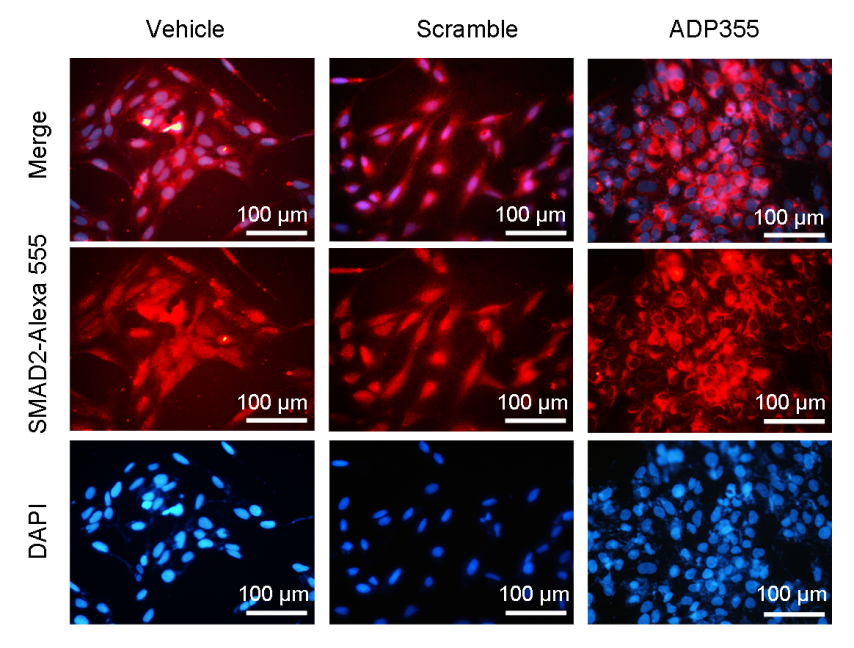


**Supplementary figure S2.** Effects of ADP355 (50ng/ml) on the nuclear translocation of SMAD2 in LX-2 cells stimulated with TGF-β1 (1ng/ml). LX-2 cells were stained for SMAD2 (red). Nuclei were stained with DAPI (blue). Ectopically expressed SMAD2 is mainly located outside of the nuclei with treatment of ADP355. Note: DAPI=4,6,-diamidino-2-phenylindole.


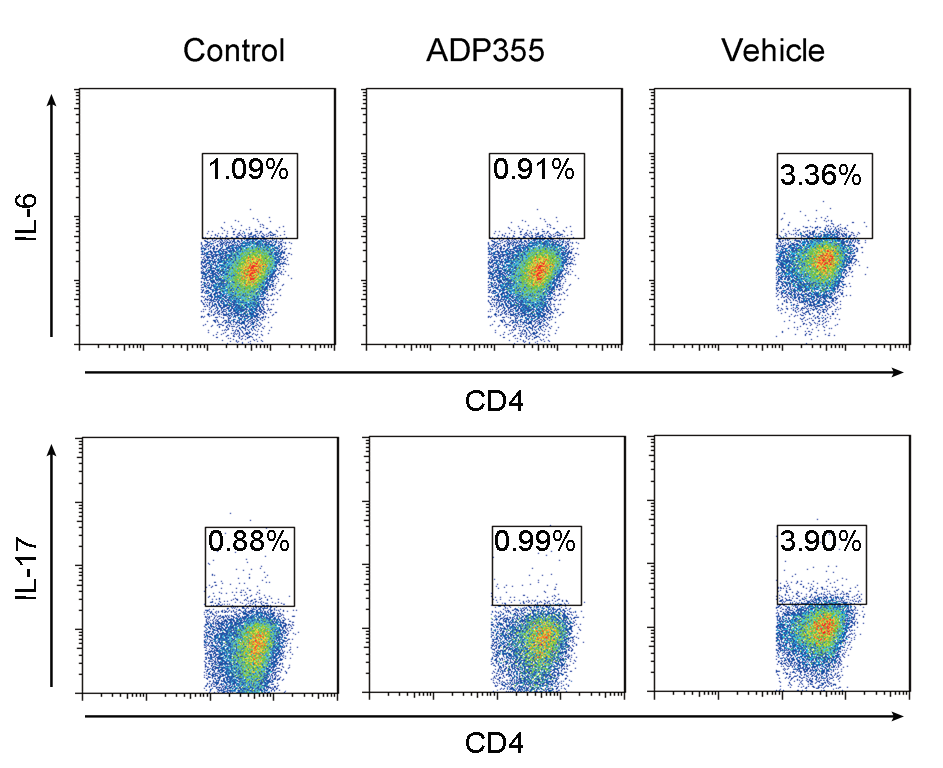


**Supplementary figure S3**. Flow cytometry analysis of helper T cells in spleens. Lymphoid fraction from mice were in vitro stimulated with phorbol-miristate acetate (PMA) (50 ng/ml), ionomycin (1 μg/ml) and brefeldin A (3 μg/ml) for 5 hours and analyzed by flow cytometry. ADP355 treatment reduces the proliferation of helper T cells induced by TAA. Representative dot plots are shown.


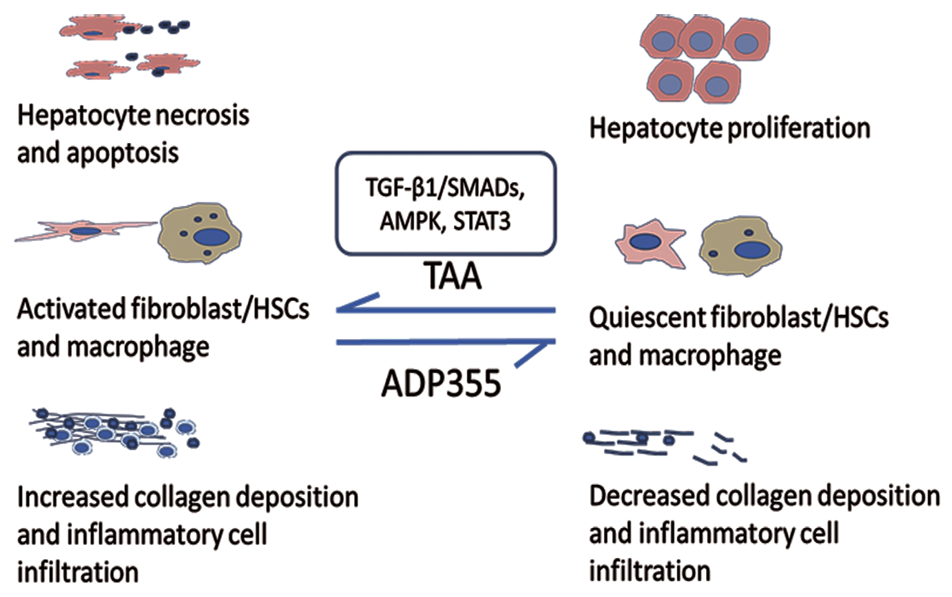


**Supplementary figure S4**. Diagram of ADP355 protective effects of on TAA-induced liver injury.
